# Prevalence, Spectrum, and Management of Thyroid Dysfunction in Children with Down Syndrome: A Retrospective Study from Southern Saudi Arabia

**DOI:** 10.3390/children13010006

**Published:** 2025-12-19

**Authors:** Youssef Ali Alqahtani, Ayed A. Shati, Ayoub Ali Alshaikh, Ali Thamer Alshahrani, Salwa Abdullah Bin Qaed, Manar Ali Alqahtani, Omar Ayidh Alotaibi, Muteb Obaid Alharthi, Mohamed Hassan Sarhan, Abdulaziz Mohammed Alrasheed, Ramy Mohamed Ghazy

**Affiliations:** 1 Department of Child Health, College of Medicine, King Khalid University, Abha 62529, Saudi Arabia; yal-qahtani@kku.edu.sa (Y.A.A.); ashati@kku.edu.sa (A.A.S.); 2 Family and Community Medicine Department, College of Medicine, King Khalid University, Abha 62529, Saudi Arabia; alashaikh@kku.edu.sa; 3 College of Medicine, King Khalid University, Abha 62529, Saudi Arabia; 441800634@kku.edu.sa (A.T.A.); 441800950@kku.edu.sa (S.A.B.Q.); 441800956@kku.edu.sa (M.A.A.); 4 College of Medicine, Shaqra University, Dawadmi 34471, Saudi Arabia; s442420367@std.su.edu.sa (O.A.A.); s442420155@std.su.edu.sa (M.O.A.); s442420115@std.su.edu.sa (A.M.A.); 5 Microbiology Section, Basic Medical Sciences Department, College of Medicine, Shaqra University, Shaqra 11961, Saudi Arabia; msarhan@su.edu.sa; 6 Medical Parasitology Department, Faculty of Medicine, Zagazig University, Zagazig 44519, Egypt; 7 Tropical Health Department, High Institute of Public Health, Alexandria University, Alexandria 61421, Egypt

**Keywords:** down syndrome, thyroid dysfunction, hypothyroidism, subclinical hypothyroidism, pediatrics, Saudi Arabia, screening adherence

## Abstract

**Highlights:**

This study found a high prevalence of thyroid dysfunction among Saudi children with Down syndrome, mainly subclinical hypothyroidism followed by autoimmune hypothyroidism. Affected children had more clinical manifestations—especially metabolic, dermatological, and gastrointestinal—than those without thyroid dysfunction. A positive family history of thyroid disease was the strongest predictor, increasing the risk more than fourfold. Despite high screening coverage and generally guideline-consistent follow-up, therapeutic management was suboptimal: around three-fourths of levothyroxine-treated patients likely needed dose adjustment. These findings highlight the need for better dose optimization, standardized diagnostic algorithms, and stronger long-term surveillance in this high-risk group.

**What are the main findings?**

- Thyroid dysfunction is very common, mainly subclinical and autoimmune hypothyroidism.
- Family history is the strongest risk factor, and most treated children need levothyroxine dose adjustment.

**What are the implications of the main findings?**

- Treatment optimization is a must to improve disease management.
- Guidelines should emphasize stricter monitoring and standardized management to close the care gap.

**Abstract:**

**Background:** Down syndrome (DS) is strongly associated with a high prevalence of thyroid dysfunction. International guidelines recommend regular screening to ensure early detection and management. This study aimed to determine the prevalence, spectrum, and management outcomes of thyroid dysfunction in children with DS in the Aseer region of Saudi Arabia. **Methods:** A retrospective cross-sectional study was conducted by reviewing the medical records of 106 children with DS from two major healthcare centers: Abha Maternity and Children Hospital and King Khalid University Medical Center. Data on demographics, clinical symptoms, thyroid function tests, autoimmune status, treatment, and follow-up patterns were collected and analyzed. **Results:** The prevalence of thyroid dysfunction was 52.8%. Subclinical hypothyroidism was the most common disorder (46.4%), followed by autoimmune hypothyroidism (30.4%). Patients with thyroid dysfunction had a significantly higher symptom burden, particularly in metabolic, dermatological, and gastrointestinal domains (*p* < 0.01). A strong family history of thyroid disease was a significant risk factor (adjusted odds ratio (aOR) = 4.57, 95% CI: 1.89–11.6, *p* < 0.001). While adherence to screening and follow-up was high (78.0% and 82.1%, respectively), a critical gap was identified in treatment optimization, with 74.4% of patients treated potentially requiring levothyroxine dose adjustment. **Conclusions:** Thyroid dysfunction is highly prevalent in the Southern Saudi children with DS, with subclinical hypothyroidism and autoimmune hypothyroidism being the most common types. Despite good screening adherence, there is a substantial need for improved treatment titration and long-term management to optimize patient outcomes in this population.

## 1. Introduction

Down syndrome (DS) is a genetic disorder that occurs when a person has a full or partial extra copy of chromosome 21 [1]. About 95% of DS cases are attributed to chromosomal non-disjunction, leading to a karyotype of 47,XX,+21 or 47,XY,+21. In roughly 2% of cases, a Robertsonian translocation, often involving chromosomes 14 and 21 and frequently of familial origin, is the cause. Additionally, mosaicism, which results from post-zygotic non-disjunction or, less frequently, trisomic rescue, accounts for another 2% of cases. The remaining 1% are due to other chromosomal rearrangements involving chromosome 21 [2,3]. DS demonstrates a strong association with advanced maternal age, with a variable live birth prevalence between 1 in 319 and 1 in 1000. A significant number of affected fetuses (50–75%) are spontaneously lost during pregnancy [4,5]. The trends in DS burden over the past three decades are heterogeneous, reflecting diverse regional and sociodemographic factors. Although global progress has been made in reducing mortality and daily adjusted life years (DALYs), the rising incidence and prevalence in some areas—particularly low- socio-demographic indices (SDI) regions—pose ongoing public health challenges [6].

DS is a multisystem disorder, and its clinical manifestations, including the severity of related health conditions and intellectual disability, can vary significantly among individuals [7]. DS is characterized by intellectual disability, distinctive facial characteristics, and high incidence of certain health conditions, such as congenital heart defects, which affect up to 50% of individuals. The most prevalent among these are atrioventricular septal defects, which are a major factor in early mortality [4]. Gastrointestinal abnormalities such as duodenal atresia and Hirschsprung disease are common, as are hematologic disorders ranging from transient newborn issues to a significantly elevated risk of leukemia [8,9,10]. Universal hypotonia contributes to motor delays, while patients also face an increased risk of seizures, early-onset Alzheimer’s disease due to Amyloid Precursor Protein (APP) gene triplication, refractive errors, and conductive hearing loss, necessitating comprehensive and lifelong specialty care [11,12,13,14]. Thanks to modern healthcare, early intervention programs, and greater social inclusion, the quality of life and life expectancy for individuals with DS have dramatically improved. With appropriate support, many people with DS now lead fulfilling lives into their 60s and beyond [15].

Thyroid dysfunction is the most common endocrine disorder found in individuals with DS, although the reported prevalence varies significantly across different populations and studies. For example, a study from the Philippines found a prevalence rate as high as 56%, primarily due to subclinical hypothyroidism [16]. Other studies reported that thyroid dysfunction is affecting an estimated 4–18% of children [17,18]. This marked variability may stem from differences in regional genetic backgrounds, rates of consanguinity [19], iodine sufficiency [20], diagnostic definitions (especially the TSH cut-off values applied) [21], access to healthcare, and compliance with screening programs, and aspects of study methodology [22]. Thyroid dysfunction is manifestations are diverse, encompassing congenital hypothyroidism, subclinical hypothyroidism, and acquired forms—both autoimmune and non-autoimmune—as well as hyperthyroidism [23]. International pediatric guidelines recommend a consistent thyroid-stimulating hormone (TSH)-based screening protocol for thyroid dysfunction in DS, starting at birth and repeated at 6 months, 12 months, and then annually [24,25,26]. If TSH is abnormal, a free thyroxine (Free T4) test should follow [27]. For patients with subclinical hypothyroidism, more frequent testing is advised [28]. Enhanced monitoring is recommended, especially for patients with positive thyroid antibody tests, as this suggests a higher likelihood of progressing to overt hypothyroidism [29].

The prevalence of DS in Saudi Arabia is 29 (95% confidence interval (CI), 22.6 to 37.1) per 100 k population which is significantly lower than developed countries like United Kingdom (83.8, 95% CI, 67.4 to 104) [30]. Understanding the context of DS in Saudi Arabia requires attention to the country’s distinct cultural, religious, and social dynamics, which collectively influence societal attitudes, access to healthcare, and the quality of life of individuals with DS and their families [31]. While Islamic values of compassion and care can foster supportive environments, persistent social stigmas may still hinder full acceptance and inclusion [32]. The Vision 2030 initiative, which aims to advance the Saudi healthcare system, is critical for enhancing early detection, intervention, and health management for conditions such as DS [33]. However, the effectiveness of these reforms ultimately depends on equitable and accessible service utilization by affected families [34].

This study addressed the critical need to evaluate and optimize real-world thyroid screening and management for children with DS in Saudi Arabia. Despite an advancing healthcare system, there is limited understanding of how international guidelines are applied locally. This research investigates the epidemiology, screening adherence, and management of thyroid dysfunction in Saudi children with DS to generate actionable, evidence-based data. Its goal is to support tailored clinical guidelines, better resource allocation, and improve long-term health and quality of life for this vulnerable population.

## 2. Materials and Methods

### 2.1. Study Design

This study employed a retrospective cross-sectional design to assess the prevalence and spectrum of thyroid dysfunction in children with DS. Saudi Arabia is divided into 13 administrative provinces. Among them, the Aseer Province, located in the south of Saudi Arabia, is governed by its capital city, Abha. With an area exceeding 81,000 km^2^ and a population of approximately two million, Aseer is a densely populated region. It is distinguished by its diverse geography and a temperate climate, situated between latitudes 17.25° N and 19.50° N and longitudes 50° E and 41° E. The study was conducted by reviewing medical records at two major healthcare centers: Abha Maternity and Children Hospital and King Khalid University Medical Center (KKUMC).

### 2.2. Study Population and Sample Size Calculation

This retrospective study included all children younger than 18 years with a confirmed DS who were being actively followed in the general pediatric clinics at the two participating healthcare centers between July 2022 and June 2025. Enrollment was explicitly not limited to patients referred to suspected thyroid disorders. The study cohort was assembled by reviewing clinic registries to identify all individuals with a documented DS diagnosis. To be eligible, participants had to be recorded as residents of the Aseer region and have at least one thyroid function test performed within the study period. The main exclusion criterion was the lack of any thyroid function test result in the medical record, or the presence of thyroid data insufficient to allow meaningful analysis. This methodology ensured that the study population represented a clinic-based group undergoing routine follow-up, rather than a sample selected based on thyroid-related complaints. The sample size for this study was determined using the OpenEpi, Version 3, open-source calculator for population-based surveys. Based on a hypothesized prevalence rate of thyroid dysfunction of 7% derived from existing literature [17], the calculation yielded a required sample size of 99 participants. This calculation assumed a 95% confidence level with a 5% margin of error and a design effect of 1. The sample size for this study was calculated a priori based on a previously reported thyroid dysfunction prevalence of approximately 7% in children with DS. Our finding of a 52.8% prevalence indicates this initial assumption was a substantial underestimate for our population. To assess the statistical adequacy of our final sample (N = 106) given this discrepancy, a post hoc power analysis was conducted using G*Power 3.1. This analysis (exact binomial test, two-tailed α = 0.05, and effect size of 0.2) confirmed the study had a power = 0.99 to detect the observed high prevalence as significantly different from the previously assumed low prevalence.

### 2.3. Data Collection Instrument

A structured data collection instrument was developed for this study to systematically gather comprehensive clinical information. The instrument covered several key domains, including demographic information (age, gender, weight, height, and body mass index (BMI)); DS characteristics (specific type); and detailed thyroid dysfunction history (diagnosis, type, and treatment). It also recorded clinical signs and symptoms, laboratory assessments (TSH, Free T4, Free T3, and antibody assays including Anti-Thyroglobulin (Anti-TG), Anti-Thyroid Peroxidase (TPO) antibodies, TSH Receptor Antibodies (TRAb)/thyroid-stimulating immunoglobulin (TSI) antibodies), as well as imaging modalities such as thyroid ultrasonography. Additional sections recorded medication history and current treatment, family history of thyroid disease, and follow-up patterns. To evaluate the patient and family perspective, the instrument integrated two specific metrics: (1) a yes/no assessment of care satisfaction (“Were you satisfied with the care your child received during today’s visit?”), and (2) a yes/no question regarding willingness to be contacted for future research studies, as documented on clinic registration forms. Furthermore, the instrument captured documented parental responses to the standardized query, “Are you aware that children with DS are at higher risk for thyroid dysfunction?” during follow-up visits.

### 2.4. Data Quality Assurance

A comprehensive data cleaning and validation process was implemented to ensure the integrity and reliability of the dataset. This process involved several key steps: missing data were systematically identified and documented to assess potential biases; inconsistent responses, particularly in categorical variables, were reviewed and standardized to maintain uniformity; outlier detection was performed on all laboratory parameters to identify and verify physiologically improbable values; and duplicate checks were conducted across participant identifiers to ensure each individual was represented only once in the final dataset.

### 2.5. Variable Coding and Transformation

- Euthyroid: Normal TSH and Free T4 for age, no history or symptoms of thyroid disease.
- Isolated hyperthyrotropinemia (IHT): Mildly elevated TSH with normal Free T4, usually asymptomatic, often transient or physiologic.
- Subclinical hypothyroidism: Elevated TSH with normal Free T4, may have mild/non-specific symptoms, often associated with autoimmune thyroiditis (i.e., Anti-TPO positive).
- Congenital hypothyroidism: Thyroid hormone deficiency present at birth, detected on newborn screening.
- Autoimmune hypothyroidism (Hashimoto’s thyroiditis): Hypothyroidism with positive Anti-TPO antibodies and/or autoimmune thyroid destruction.
- Graves’ disease: Hyperthyroidism due to TRAb/TSI, with clinical features of hyperthyroidism.
- Other/Overt hyperthyroidism: Hyperthyroidism not caused by Graves’ disease (e.g., toxic nodule, toxic multinodular goiter, thyroiditis).
- Secondary (Central) hypothyroidism: Low FT4 with low or inappropriately normal TSH due to pituitary or hypothalamic dysfunction.

#### 2.5.1. Symptom Coding

To facilitate quantitative analysis of clinical presentations, reported symptoms were systematically encoded into structured variables. First, binary indicator variables (e.g., fatigue, constipation) were created for each documented symptom, with a value of 1 indicating presence and 0 indicating absence. The variable symptom weight changes were derived through a targeted text analysis of clinical notes. Subsequently, these indicators were aggregated into several composite symptom domain scores to capture the overall burden and pattern of symptomatology. The following scores were calculated:

- Total symptom score: The sum of all individual symptom indicators, representing the overall symptom burden.
- Metabolic symptom score: A composite of fatigue, weight changes, and cold intolerance.
- Neurodevelopmental symptom score: A composite of developmental delay and behavioral changes.
- Dermatological symptom score: Represented by the presence of dry skin.
- Gastrointestinal symptom score: Represented by the presence of constipation.

#### 2.5.2. Comorbidity Assessment

To systematically characterize the complex health profile of the study population, all documented comorbid conditions were translated into structured quantitative variables. This was achieved by creating binary indicator variables (coded as 1 for presence and 0 for absence) for each major comorbidity. The assessed conditions included congenital heart disease, autoimmune disorders, type 1 and type 2 diabetes mellitus, leukemia, celiac disease, and asthma, in addition to other clinically significant conditions documented within the medical records. These indicators were then aggregated to create composite measures of disease burden. A total comorbidity score was calculated as the sum of all individual comorbidity indicators to represent the overall multimorbidity load.

#### 2.5.3. Laboratory Tests

Thyroid function and autoimmune status were classified according to established pediatric clinical reference ranges and corresponding laboratory findings.

TSH reference classification by age group [21]:

- 0–1 month: 0.7–18.1 mU/L.
- 1–12 months: 1.12–8.21 mU/L.
- 1–5 years: 0.80–6.26 mU/L.
- 6–10 years: 0.80–5.40 mU/L.
- 11–14 years: 0.70–4.61 mU/L.
- 15–18 years: 0.50–4.33 mU/L.

Free T4 classification: FT4 reference intervals (ng/dL) [35]:

- 1 month to <1 year: 1.3–2.8.
- 1 to <3 years: 1.3–2.4.
- 3 to <8 years: 1.3–2.4.
- 8 to <18 years: majority 1.3–2.4.

Autoimmune status classification: Autoimmune thyroid status was determined based on Anti-TPO and Anti-TG antibody results:

- Autoimmune positive: Elevated level of either Anti-TPO or Anti-TG antibody.
- Autoimmune negative: Normal levels of both Anti-TPO and Anti-TG antibodies.
- Not tested: Absence of results for both antibody tests.
- Partially tested: Only one of the two antibody tests was performed.

#### 2.5.4. Care Gap Identification

##### Caregivers’ Perspective and Awareness-Care Gap

The caregivers’ perspective was assessed through two metrics. First, care satisfaction and willingness to participate in future research. Furthermore, to identify an awareness-care gap, parents were systematically asked during follow-up if they were aware that children with DS are at higher risk for thyroid dysfunction, as documented in records. It was defined as an affirmative response to this awareness question concurrent with documented dissatisfaction with the provided thyroid-related care or information.

##### Screening Adherence (For Non-Thyroid Patients)

For individuals without known thyroid dysfunction, adherence to recommended annual thyroid screening was classified as follows:

- Adherent: Last thyroid screen conducted within ≤15 months, allowing a 3-month grace period beyond the annual recommendation.
- Moderately adherent: Last thyroid screen conducted 15–24 months ago.
- Non-adherent: Last thyroid screen conducted >24 months ago.

##### Follow-Up Adequacy Assessment

For individuals with known thyroid dysfunction, the adequacy of clinical follow-up was evaluated based on established guidelines for thyroid monitoring in DS. Adherence was defined according to the following criteria [22,36,37]:

- Severe or moderate thyroid dysfunction, e.g., overt hypothyroidism, hyperthyroidism: Appropriate follow-up: ≤6 months
- Mild thyroid dysfunction: (e.g., subclinical hypothyroidism): Appropriate follow-up: ≤12 months
- Patients whose follow-up intervals exceeded these timeframes were classified as having inadequate monitoring.

##### Biochemical Improvement

For participants receiving levothyroxine, treatment adequacy was defined by the achievement of biochemical euthyroid. This was assessed using serum TSH levels. For patients with central (secondary) hypothyroidism, where TSH is an unreliable marker, adequacy was defined by a normal FT4 level. Age-specific reference ranges for TSH and FT4 were applied based on our institutional laboratory standards.

### 2.6. Statistical Analysis Plan

Descriptive statistics were employed to summarize the study data. Continuous variables were presented as mean ± standard deviation for normally distributed data or median with interquartile range (IQR) for non-normal distributions. This determination was made using Shapiro–Wilk tests and visual inspection of histograms and Q-Q plots. Categorical variables were summarized using frequencies and percentages. All analyses were stratified by thyroid dysfunction status (“yes” or “no”) to enable comparative assessment across patient groups. For analytical comparisons, the chi-square test was employed to analyze associations between categorical variables. When the assumptions of the chi-square test were not met, Fisher’s exact test was utilized instead. A multivariable logistic regression analysis was performed to identify factors independently associated with thyroid dysfunction. The results of this analysis are summarized in a forest plot, which presents the strength of associations as adjusted odds ratios (aOR) with corresponding 95% CI. Model fit and predictive accuracy were assessed using the Hosmer-Lemeshow goodness-of-fit test. The analysis was conducted using R statistical programming language (R version 4.3.1 (2023-06-16 ucrt)) within the R Studio integrated development environment. Key packages included tidyverse for data manipulation and visualization, lubridate for date-time operations, openxlsx/writexl for Excel file handling, and janitor for data cleaning utilities. Throughout the analysis, robust data management procedures were applied, encompassing complete documentation of every data transformation, strict version control for all code, and structured data validation checks at each processing step to safeguard reproducibility and uphold analytical integrity.

### 2.7. Ethical Considerations

This study was carried out in full compliance with established ethical standards for research involving human participants. The research protocol was formally approved by the Institutional Review Board (IRB) at King Khalid University (ECM#2024-4708). To safeguard participant confidentiality, a stringent data anonymization process was employed, ensuring that no direct personal identifiers (including names, medical record numbers, or contact details) were retained in the dataset used for analysis. All data handling and storage followed robust security procedures, such as encryption of electronic files and restricted-access systems, in accordance with institutional data protection regulations. Because this was a retrospective study, the ethics committee granted a waiver of informed consent. The principle of appropriate secondary data use was respected by limiting the analysis strictly to the predefined research aims. Furthermore, all study results are reported only in aggregated form, so that no individual participant can be identified.

## 3. Results

We retrieved data for 123 children with DS from clinic registries. Seventeen were excluded due to insufficient thyroid function data (i.e., missing both TSH and F T4 results), yielding a final analytical cohort of 106 patients, aged 1.9 to 17 years, who had undergone thyroid screening as part of routine care. Of these, 59 (55.7%) were female and 47 (44.3%) and 89.6% had trisomy 21. The cohort was predominantly symptomatic, with 98.1% (104/106) of patients presenting with at least one symptom, and over half (53.5%) reporting two or more. A substantial proportion of participants had comorbidities, with 63.2% (67/106) having at least one. Additionally, 46.2% had a positive family history of thyroid disease, and the majority (71.7%) were already aware of their high risk for thyroid dysfunction Table 1.

**Figure 1:**
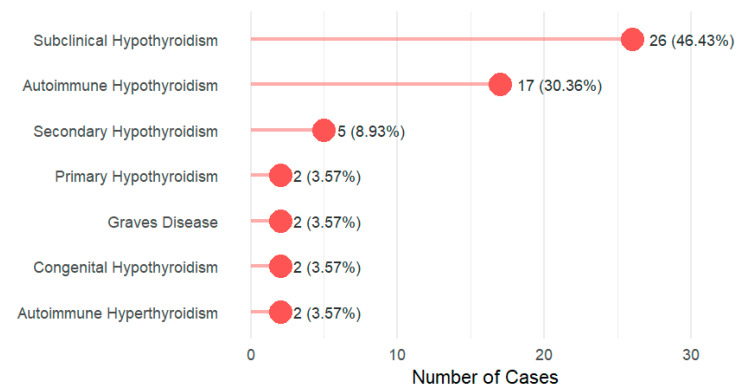
Distribution of thyroid dysfunction types among the study participants (N = 56).

The prevalence of thyroid diseases among DS cases was 52.8% (56/106). The age, gender, and family history are presented in Supplementary Table S1. Subclinical hypothyroidism emerges as the most prevalent disorder, representing nearly half of all cases (46.43%, n = 26), followed by autoimmune hypothyroidism at 30.36% (n = 17). Figure 1. Prevalence of thyroid dysfunction by DS karyotype is described in Supplementary Table S2.

Patients with thyroid dysfunction showed a male predominance (53.6% male vs. 46.4% female; *p* = 0.043). A clear gradient in symptom burden was observed between groups (*p* = 0.005). Patients without thyroid dysfunction predominantly report 0–2 symptoms (88.0% fall within this range), while those with thyroid dysfunction demonstrated higher symptom counts, with 28.6% reporting 3 symptoms and 10.8% reporting 4–6 symptoms. Across symptom domains, patients with thyroid dysfunction exhibited significantly greater metabolic (*p* = 0.0058), dermatological (*p* = 0.0063), and gastrointestinal (*p* < 0.0001) manifestations, although neurodevelopmental symptoms did not differ significantly (*p* = 0.1531). Biochemical markers also diverged between groups: the TSH level was substantially higher among those with thyroid dysfunction (median 6.0 (4.6, 7.6) vs. 4.0 (3.0, 4.2); *p* < 0.001), while fT4 levels were significantly lower (median 13.9 (12.0, 15.6) vs. 14.0 (13.0, 16.0); *p* = 0.02). A family history of thyroid disease showed a strong association with dysfunction (*p* < 0.001), with 66.1% of affected patients reporting a positive family history compared to only 24.0% in the non-dysfunction group. Several characteristics showed no statistically significant differences between groups, including age distribution (*p* = 0.2), total comorbidities (*p* = 0.6), height (*p* = 0.2), weight (*p* = 0.077), and BMI (*p* = 0.4). Awareness of being at high risk was also not significantly different (*p* = 0.1) Table 2.

Family history of thyroid disease demonstrates the strongest association with thyroid disorders among children with DS, with an odds ratio of 4.57 (95% CI: 1.89–11.6, *p* < 0.001). The analysis also revealed a statistically significant association between total symptoms and thyroid dysfunction, with each additional symptom corresponding to a 92% increase in odds (aOR = 1.92, 95% CI: 1.22–3.25, *p* = 0.008) Table 3.

Most caregivers of children with thyroid dysfunction (82.1%) demonstrated strong adherence to thyroid follow-up and monitoring guidelines, and 73.2% had their autoimmune status fully tested (see Supplementary Tables S3–S6 and Supplementary Figure S1). However, regarding treatment, only 26.8% of patients on levothyroxine (n = 11/41) were found to be on an appropriate dose, with the majority (73.2%) potentially requiring adjustment (for details on appropriate dosing across types of thyroid dysfunction, see Supplementary Table S7). From the patient and caregiver perspective, a high proportion (78.6%) were aware of the associated thyroid risk. Despite this awareness, 30.4% reported dissatisfaction with care, resulting in a notable awareness-care gap where 31.8% of aware caregivers were nonetheless dissatisfied. In contrast, engagement with research was very strong, with 92.9% of participants willing to participate in future studies Table 4.

Among caregivers of children without thyroid dysfunction (n = 50), adherence to thyroid screening guidelines was strong, with the majority (78.0%) being adherent to annual screening within a 15-month period. In this cohort, 64% were aware of their high thyroid risk. Of these, 12.0% reported dissatisfaction with their thyroid care, resulting in an awareness-care gap—patients who were both aware and dissatisfied—of 6.0% overall. When calculated among only the aware patients, the prevalence of the awareness-care gap was 9.4% (3/32). Statistical analysis using Fisher’s exact test indicated there was no statistically significant association between awareness and dissatisfaction in this cohort (*p* = 0.654). In contrast, willingness to participate in future research was very high, with 94.0% of patients agreeing to be contacted for future studies Table 5.

## 4. Discussion

DS is associated with several medical conditions, one of which is thyroid dysfunction. Patients with DS have a higher risk of thyroid function deterioration over time, a progression that appears to be associated with elevated baseline TSH levels at diagnosis and the presence of thyroid autoimmunity [38]. In this study, we aimed to highlight the prevalence of thyroid disorders among DS and describe the clinical management in Saudi Arabia.

### 4.1. Summary of the Main Findings

This study of 106 Saudi children with DS revealed a strikingly high prevalence of thyroid dysfunction at 52.8%. The spectrum of dysfunction was dominated by subclinical hypothyroidism (46.4%) and autoimmune hypothyroidism (30.4%). Patients with thyroid dysfunction exhibited a significantly higher clinical symptom burden, particularly in the metabolic, dermatological, and gastrointestinal domains. A positive family history of thyroid disease emerged as the strongest independent risk factor, increasing the odds of thyroid disease by more than 4.5-fold (aOR = 4.57). While adherence to screening and follow-up was excellent (78.0% and 82.1%, respectively), a critical gap was identified in treatment optimization. Among children receiving levothyroxine, 74.4% were on a potentially non-ptimal dose, highlighting a significant disparity between effective detection and adequate therapeutic management.

### 4.2. Interpretation of the Main Findings

Prevalence of thyroid diseases among DS: In this study, we found that more than half of the participants had thyroid dysfunction. Similarly, a previous study reported that 77 out of 133 individuals (57.9%) were affected by thyroid dysfunction [39]. This prevalence was notably higher than that reported in a previous study conducted in South Africa, where thyroid dysfunction was identified in 34.5% of participants [40]. It also exceeded findings from a study in Ethiopia, which documented a prevalence of 47.7% [41]. Furthermore, our prevalence was higher than those reported in international studies from developed settings, such as the United States, where a prevalence of 24% to 32.5% was observed [23,42]. The considerable variation in reported thyroid abnormalities among individuals with DS likely stems from several factors, including differences in diagnostic criteria, study sample sizes, age distributions, and laboratory methods for assessing thyroid function [43]. Critically, lower prevalence figures in some studies may reflect inadequate screening practices rather than a genuinely lower disease burden. For example, data from a large United States multi-institutional registry indicated that only 47.7% of patients were up-to-date on thyroid screening upon presentation to a specialty clinic; among those actually tested, 19.0% received a new diagnosis [44]. This pattern closely corresponds to our own experience. The high prevalence observed in our cohort was directly contingent upon its excellent screening adherence rate of 78.0%. Collectively, these findings underscore a key paradox: while adherence to screening guidelines is the cornerstone of early identification, systematic screening itself remains inconsistently applied in real-world settings. Our data therefore reinforces the essential role of dedicated DS clinics and structured care pathways. Such models are critical for closing the screening gap and thereby uncovering the true—and significant—burden of thyroid dysfunction in this population.

Type of thyroid dysfunction: In the current study, subclinical hypothyroidism was the most common thyroid disorder in this cohort, followed by autoimmune hypothyroidism, a pattern consistent with the existing literature [42,45]. Notably, we observed a high burden of autoimmunity. Among patients with subclinical hypothyroidism who were tested for thyroid antibodies, 17 out of 21 (81.0%) were positive for anti-TPO or anti-TG antibodies (see Supplementary Files). This finding underscores the central role of thyroid autoimmunity in driving thyroid dysfunction in DS, a mechanism potentially linked to interferon overexpression encoded on chromosome 21 [46,47]. Autoimmune thyroid disease in DS presents a distinct phenotype. Children with DS show increased susceptibility to both Hashimoto’s thyroiditis and Graves’ disease, independent of typical risk factors, and have higher rates of associated extra-thyroidal autoimmune disorders [48]. Crucially, this autoimmunity has direct prognostic implications. A prospective multicenter study by Pepe et al. [49] found that autoimmune subclinical hypothyroidism in DS children was associated with a significantly higher five-year progression rate to overt hypothyroidism compared to non-autoimmune cases (35.1% vs. 17.2%), with autoimmunity and higher baseline TSH as independent predictors. Therefore, our finding of widespread antibody positivity, combined with this evidence, underscores that subclinical hypothyroidism in DS is frequently autoimmune in nature and carries a substantial risk of progression. This necessitates vigilant, specialized monitoring and underscores the need for tailored follow-up protocols that account for autoimmune status and baseline TSH levels.

In this study, congenital hypothyroidism was diagnosed among approximately 2% of the participants. In the general population, congenital hypothyroidism—one of the most common preventable causes of intellectual disability—is detected in 1 in 2000 to 3000 live births through neonatal screening. [50] The prevalence of congenital hypothyroidism in children with DS is estimated to be 28–35 times higher than in the general population [18]. The markedly higher prevalence of congenital hypothyroidism in children with DS underscores the critical need for early and routine thyroid screening in this population. Timely identification and treatment of congenital hypothyroidism can prevent intellectual disability and optimize developmental outcomes, highlighting the importance of vigilant monitoring beyond standard neonatal screening protocols.

### 4.3. Factors Associated with Thyroid Dysfunction

Age: In this study, the median age at diagnosis of thyroid dysfunction in children with DS was 8.4 years (IQR: 6.2–10.4) and age itself was not a significant predictor of thyroid disorders in our cohort. This finding stands in contrast to the established screening schedule recommended by the American Academy of Pediatrics (AAP), which begins at birth, with follow-up at 6 months, 12 months, and annually thereafter [23,42]. The delayed diagnosis occurred despite the presence of early screening initiatives in Saudi Arabia. This pattern indicates that while congenital hypothyroidism is a well-recognized concern, the clinically detected onset of thyroid dysfunction in DS frequently occurs in later childhood rather than in infancy. Therefore, our data reinforce that screening must be sustained and lifelong to detect these later-onset cases.

Gender: In this study, gender was significantly associated with thyroid disorders, with male children showing a higher prevalence than females. This finding contrasts with patterns observed in non-DS populations, where females typically exhibit higher rates of thyroid dysfunction [51,52]. However, this association was no longer significant after multivariable regression analysis. A similar finding was reported by Maphumulo et al. [39], while Amr [18] reported no gender differences in thyroid dysfunction among children with DS.

Family history of thyroid diseases: In this study, a family history of thyroid disease was a strong predictor of thyroid dysfunction. A similar finding was reported by Al Qassimi et al., [53] who observed a significant association between a family history of endocrinopathies and various forms of hypothyroidism. Moreover, Corona-Rivera et al., [54] reported that in children with DS, a family history of hypothyroidism may increase the risk of congenital hypothyroidism by up to eightfold. In contrast, other studies have reported lower rates of positive family history [18,55]. These findings highlight the importance of obtaining detailed family history when assessing children with DS. Early identification of those at higher risk could guide more targeted and timely thyroid screening, allowing for earlier diagnosis and intervention to prevent complications associated with thyroid dysfunction.

Symptoms severity: In the present study, patients with DS and thyroid abnormalities demonstrated a significantly greater overall symptom burden compared to their euthyroid counterparts. This finding is critically important precisely because the classic clinical features of hypothyroidism—such as fatigue, weight gain, and constipation—substantially overlap with the baseline phenotypic characteristics of DS itself, rendering them unreliable as specific diagnostic indicators for any individual patient [18]. This overlap creates a diagnostic masking effect: while thyroid dysfunction measurably exacerbates the somatic and cognitive manifestations of DS [56,57], the resulting worsening is often attributed solely to the underlying syndrome. Thyroid hormone affects virtually every organ system, including the cardiovascular, central nervous, and metabolic systems; thus, untreated dysfunction can silently compound the intrinsic health challenges in this population. Our data therefore resolve a key clinical paradox: significant thyroid-related morbidity can be present yet clinically obscured. This underscores the indispensable role of protocol-driven, lifelong biochemical screening—rather than reliance on symptom recognition—to ensure the early detection and management of this systemic, treatable condition in children with DS.

### 4.4. Awareness Gap

This study revealed a significant awareness–care gap affecting 9.4% of parents with euthyroid children who have DS and 31.8% of parents with children experiencing thyroid dysfunction. This suggests that the main issue is not the initial diagnosis itself but rather the lack of continuity in care following the diagnosis. This gap has serious consequences, as thyroid hormones are vital for neurodevelopment and metabolic balance [58]; inconsistent management can worsen the cognitive and physical challenges associated with DS. Additionally, symptoms of hypothyroidism often resemble those of DS, which can lead to a misinterpretation of clinical deterioration because of the syndrome rather than inadequate thyroid management, perpetuating a harmful cycle of undertreatment. Therefore, our findings suggest a need for a change in clinical priorities. While regular screening is essential for timely detection, it is not sufficient on its own. It is crucial to ensure that a diagnosis is paired with ongoing, accessible care [57]. This requires well-organized follow-up procedures, patient education, and care coordination—preferably through specialized DS clinics—to effectively close the gap between awareness and satisfactory long-term management.

Management Gap: In this study, we observed that thyroid ultrasound was not carried out in a substantial proportion of affected children, and most of them received inappropriate levothyroxine therapy. Several factors are likely to contribute to the observed suboptimal biochemical control among levothyroxine-treated patients. First, physiological and age-related variations in thyroid hormone requirements, the influence of comorbid conditions, and polypharmacy can complicate dose titration and stability [59]. Additionally, children with DS are often managed by multiple subspecialties [17], which—in the absence of integrated, multidisciplinary clinics or standardized pediatric endocrinology referral pathways—may result in fragmented thyroid care, ambiguous monitoring responsibilities, and the deprioritization of thyroid management amid competing clinical demands. Finally, health system barriers, including limited timely access to laboratory services, clinical staff turnover, and the lack of automated reminders for follow-up testing, can collectively contribute to monitoring lapses and delayed treatment adjustments [22]. These interconnected clinical, structural, and logistical factors underscore the need for systematic approaches to optimize long-term thyroid management in this population.

### 4.5. Strengths and Limitations

This study possesses a notable strength, including its focus on an under-investigated population in Saudi Arabia. However, the findings must be interpreted in light of certain limitations, primarily the retrospective cross-sectional design, which relies on the accuracy and completeness of medical records and cannot establish causality. The relatively modest sample size from two centers in a single region may limit the generalizability of the results to the broader national or international DS population. Furthermore, there is substantial symptom ascertainment bias: A key weakness of this retrospective approach is its dependence on unstructured clinical notes to identify symptoms. Clinical features that overlap between thyroid dysfunction and DS—such as constipation, fatigue, and dry skin—are probably under-documented in routine records and highly susceptible to variability in how clinicians record them. This creates a considerable risk of under-ascertainment and non-differential misclassification, markedly undermining the credibility of the reported symptom prevalence and constraining our capacity to robustly link symptoms to thyroid dysfunction status.

Implications of the study: The findings highlight a critical need to bridge the gap between effective screening and optimal treatment management for thyroid dysfunction in children with DS. While the high observed prevalence and strong familial risk reinforce the necessity of vigilant, guideline-driven annual screening, the widespread suboptimal levothyroxine dosing reveals a substantial opportunity for quality improvement in patient care. Consequently, clinical protocols must evolve beyond detection to emphasize systematic treatment titration, leveraging dose calculation and regular biochemical follow-up. Furthermore, these results advocate for the development of localized guidelines and educational initiatives tailored to the Saudi population, ensuring that robust screening translates directly into improved long-term health outcomes for this vulnerable group.

## 5. Conclusions

This study demonstrates that thyroid dysfunction is highly prevalent among Saudi children with DS, with a distinct spectrum dominated by subclinical and autoimmune hypothyroidism. The identification of a strong family history as a key risk factor and the significant correlation between thyroid dysfunction and a higher burden of clinical symptoms underscore the substantial impact of this comorbidity. While the findings reveal robust adherence to international screening and monitoring guidelines within the studied healthcare centers, they also expose a critical gap in the management phase, specifically in the optimization of levothyroxine therapy. This disparity between effective detection and suboptimal treatment titration highlights a crucial area for quality improvement. Therefore, to fully translate the benefits of early screening into improved long-term outcomes, clinical efforts must be intensified to not only maintain vigilant monitoring but also to ensure precise and individualized treatment for this vulnerable patient population.

## Figures and Tables

**Table 1:** Baseline demographic and clinical characteristics of the study cohort with down syndrome (N = 106).

Variable	Characteristic	Overall N = 106
Gender	Female	59.0 (55.7%)
	Male	47.0 (44.3%)
Age (years)	Median (Q1, Q3)	8.0 (5.9, 10.7)
	Min, Max	1.9, 17.0
Type of DS	Trisomy 21	95 (89.6%)
	Translocation	6 (5.6%)
	Mosaic	5 (4.7%)
Total symptoms	0	2.0 (1.9%)
	1	39.0 (36.8%)
	2	37.0 (34.9%)
	3	20.0 (18.9%)
	4	4.0 (3.8%)
	5	2.0 (1.9%)
	6	2.0 (1.9%)
Total comorbidities	0	39.0 (36.8%)
	1	52.0 (49.1%)
	2	15.0 (14.2%)
TSH level	Median (Q1, Q3)	4.4 (3.4, 6.0)
	Min, Max	0.1, 19.0
Free T4	Mean ± SD	14.2 ± 2.5
	Min, Max	9.8, 22.0
Height (cm)	Mean ± SD	122.0 ± 23.8
	Min, Max	14.0, 162.0
Weight (kg)	Mean ± SD	40.5 ± 17.4
	Min, Max	12.0, 100.0
Body mass index	Median (Q1, Q3)	25.4 (22.4, 28.4)
	Min, Max	15.0, 46.5
Ultrasound performed		4 (3.8%)
Family history of thyroid disease		49.0 (46.2%)
Awareness of high risk of thyroid disease		76.0 (71.7%)

**Table 2:** Clinical, biochemical, and symptom profiles of patients with and without thyroid dysfunction.

Characteristic	Category	No Thyroid Dysfunction (n = 50)	With Thyroid Dysfunction (n = 56)	*p*-Value
Gender	Female	33 (66.0%)	26 (46.4%)	0.043
	Male	17 (34.0%)	30 (53.6%)	
Age (years)	Median (Q1, Q3)	7.4 (5.4, 11.0)	8.4 (6.2, 10.4)	0.2
	Min, Max	2.2, 15.0	1.9, 17.0	
Total symptoms	0	2 (4.0%)	0	0.005
	1	25 (50.0%)	14 (25.0%)	
	2	17 (34.0%)	20 (35.7%)	
	3	4 (8.0%)	16 (28.6%)	
	4	2 (4.0%)	2 (3.6%)	
	5	0	2 (3.6%)	
	6	0	2 (3.6%)	
Metabolic symptoms	Median (IQR)	1 (0–1)	1 (1–1)	0.0058
Neurodevelopmental symptoms	Median (IQR)	0 (0–1)	0 (0–1)	0.2
Dermatological symptoms	n (%)	4/50 (8%)	17/56 (30.4%)	0.0063
Gastrointestinal symptoms	n (%)	16/50 (32%)	51/56 (91.1%)	<0.0001
Total Comorbidities	0	21 (42.0%)	18 (32.1%)	0.6
	1	23 (46.0%)	29 (51.8%)	
	2	6 (12.0%)	9 (16.1%)	
TSH Level	Median (Q1, Q3)	4.0 (3.0, 4.2)	6.0 (4.6, 7.6)	<0.001
	Min, Max	1.9, 5.1	0.0, 19.0	
Free T4	Median (Q1, Q3)	14.0 (13.0, 16.0)	13.9 (12.0, 15.6)	0.02
	Min, Max	11.0, 19.0	9.8, 22.0	
Height (cm)	Median (Q1, Q3)	126.5 (107–135)	129.0 (119–139.5)	0.2
	Min, Max	70–159	65–162	
Weight (kg)	Median (Q1, Q3)	35.0 (24–47)	39.5 (32–49)	0.077
	Min, Max	14–86	12–100	
Body mass index	Median (Q1, Q3)	25.4 (21–28.3)	25.5 (22.6–28.6)	0.4
	Min, Max	15–46.5	17.1–44	
Family history of thyroid disease	No	38 (76.0%)	19 (33.9%)	<0.001
	Yes	12 (24.0%)	37 (66.1%)	
Awareness that children with down syndrome are at higher risk for thyroid dysfunction	No	18 (36.0%)	12 (21.4%)	0.1
	Yes	32 (64.0%)	44 (78.6%)	

**Table 3:** Predictors of thyroid dysfunction among children with Down syndrome.

Characteristic	OR	95% CI	*p*-Value
Gender			
Female	-	-	
Male	1.70	0.69, 4.24	0.2
Total symptoms	1.92	1.22, 3.25	0.008
Family history of thyroid disease			
No	-	-	
Yes	4.57	1.89, 11.6	<0.001

Abbreviations: CI = Confidence Interval, OR = Odds Ratio.

**Table 4:** Summary of screening, monitoring, clinical status, and patient perspective.

Category	Variable	Value/Description	Count (n)	Percentage (%)
Follow-up and Monitoring	Appropriate follow-up	Appropriate	46	82.1%
		Inadequate	10	17.9%
	Appropriate dosing of levothyroxine (n = 41) *	May need adjustment	30	73.2%
		Appropriate	11	26.8%
Clinical Status	Autoimmune testing	Fully tested	41	73.2%
		Partially tested	4	7.1%
		Not tested	11	19.6%
Patient and Caregiver Perspective	Care satisfaction	Dissatisfied	17	30.4%
	Aware of high thyroid risk	Aware	44	78.6%
	Awareness-care gap	Aware but not satisfied	14	25.0%
	Awareness-care gap prevalence	(Among aware patients, n = 44)	14	31.8%
	Willing to participate in future studies		52	92.9%

*: This analysis includes only the 41 patients from the total thyroid disorder cohort (n = 56) who were prescribed levothyroxine. Chi-square test of independence revealed no statistically significant association between awareness of high thyroid risk and satisfaction with thyroid care (χ^2^(1) = 1.70, *p* = 0.156).

**Table 5:** Screen adherence in patients without thyroid dysfunction.

Metric		Category	n	%
Screening Adherence		Total patients without thyroid dysfunction	50	100%
		Adherent (≤15 months)	39	78.0%
		Moderately Adherent (15–24 months)	6	12.0%
		Non-Adherent (>24 months)	5	10.0%
Patient and Caregiver Perspective	Awareness and Satisfaction	Aware of high thyroid risk	32	64.0%
		Dissatisfied with thyroid care	6	12.0%
		Awareness-care gap (Aware but dissatisfied)	3	6.0%
	Awareness-care gap prevalence	Among aware patients (n = 32)	3	9.4%
	Willing to participate in future studies		47	94.0%

## Data Availability

The data presented in this study are available in [Down ] at [https://1drv.ms/x/c/17beb82f5043a719/IQB7XEdD4IjQTbNtz8RyqlsxAS0_3EpBmU3n1lnyvZcwvZg?e=drRbdD].

## Supplementary Materials

The following supporting information can be downloaded at: https://www.mdpi.com/article/10.3390/children13010006/s1, Figure S1: Number and percentage of patients with thyroid disease tested (partially or fully) or no tested for Anti-TPO and Anti-TG.; Table S1: Demographic and Clinical Characteristics of Participants by Type of Thyroid Dysfunction; Table S2: Prevalence of Thyroid Dysfunction by Down Syndrome Karyotype; Table S3: Anti-Thyroglobulin (Anti-TG) Antibody Status by Thyroid Dysfunction Type; Table S4: Anti-Thyroid Peroxidase (TPO) Antibody Positivity by Thyroid Dysfunction Type in Children with Down Syndrome; Table S5: Thyroid Autoantibody Positivity by Thyroid Dysfunction Type and Down Syndrome Karyotype; Table S6 : Thyroid Autoantibody Status by Down Syndrome Karyotype; Table S7: Treatment Adequacy by Thyroid Dysfunction Type; Figure S2: Forest plot of the predictors of having thyroid dysfunction among patients with DS.

## Abbreviation

**Abbreviation**	**Full Term**
AAP	American Academy of Pediatrics
Anti-TG	Anti-Thyroglobulin Antibody
Anti-TPO	Anti-Thyroid Peroxidase Antibody
BMI	Body Mass Index
CI	Confidence Interval
DALYs	Disability-Adjusted Life Years
DS	Down Syndrome
FT4	Free Thyroxine
IRB	Institutional Review Board
IQR	Interquartile Range
OR	Odds Ratio
SCH	Subclinical Hypothyroidism
SD	Standard Deviation
TRAb	TSH Receptor Antibodies
TSH	Thyroid-Stimulating Hormone
CHD	Congenital Heart Disease
FT4/Free T4	Free Thyroxine
SDI	Socio-Demographic Index
TD	Thyroid Dysfunction
