# Supplementary material for: Prevalence, Spectrum, and Management of Thyroid Dysfunction in Children with Down Syndrome: A Retrospective Study from Southern Saudi Arabia
Source: Children (Basel). 2025 Dec 19;13(1):6. doi: 10.3390/children13010006 (PMC12840509; doi:10.3390/children13010006)
Supplement: Supplementary file 1 [file children-13-00006-s001.zip › children-4032646-supplementary.pdf]

A total of 56 pediatric patients with Down syndrome had thyroid dysfunction. Subclinical hypothyroidism (n=26) was the most common diagnosis. Patients present at various ages, with Secondary Hypothyroidism (n=5) being the earliest at a mean of 4.6 years, and primary hypothyroidism (n=2) the latest at 15.0 years. A positive family history was highly prevalent across all groups, especially in the largest cohorts: 57.7% (15/26) in subclinical and 70.6% (12/17) in autoimmune hypothyroidism (Supplementary Table S1).

Supplementary Table S1: Demographic and Clinical Characteristics of Participants by Type of Thyroid Dysfunction.

Thyroid Type	N	Age (years)	Female (n/N, %)	Family History (n/N, %)
Subclinical Hypothyroidism	26	8.6 ± 3.4	12/26 (46.2%)	15/26 (57.7%)
Autoimmune Hypothyroidism	17	9.4 ± 3.0	7/17 (41.2%)	12/17 (70.6%)
Secondary Hypothyroidism	5	4.6 ± 2.1	3/5 (60.0%)	3/5 (60.0%)
Autoimmune Hyperthyroidism	2	12.2 ± 6.8	2/2 (100.0%)	2/2 (100.0%)
Congenital Hypothyroidism	2	7.0 ± 0.1	1/2 (50.0%)	2/2 (100.0%)
Graves' Disease	2	11.1 ± 1.4	0/2 (0.0%)	2/2 (100.0%)
Hypothyroidism	2	15.0 ± 2.8	1/2 (50.0%)	1/2 (50.0%)

Among the 106 children with DS, those with standard trisomy 21 (n=95) showed the highest thyroid dysfunction prevalence at 53.7% (51/95). Children with translocation (n=6) and mosaic (n=5) karyotypes showed prevalence rates of 50.0% and 40.0%, respectively. Statistical testing confirmed no significant association between DS karyotype and likelihood of thyroid dysfunction (p=0.829), indicating that the elevated thyroid risk applies across all genetic subtypes of DS (Supplementary Table S2).

Supplementary Table S2: Prevalence of Thyroid Dysfunction by Down Syndrome Karyotype.

Down Syndrome Type	No Thyroid Disorder n (%)	With Thyroid Disorder n (%)	Total n (%)	Thyroid Disorder Prevalence (%)
Trisomy 21	44 (46.3%)	51 (53.7%)	95 (100%)	53.7%
Translocation	3 (50.0%)	3 (50.0%)	6 (100%)	50.0%
Mosaic	3 (60.0%)	2 (40.0%)	5 (100%)	40.0%

$p \approx 0.829$

Autoimmune hypothyroidism had 88.2% of cases being fully tested. Autoimmune hyperthyroidism (n=2) and Graves' Disease (n=2) showed 100% full testing. Congenital

hypothyroidism (n=2) is split evenly between not tested and fully tested. Primary hypothyroidism (n=2) shows 100% not tested. Secondary hypothyroidism (n=5) includes 4 fully tested (80%) and 1 not tested (20%). Subclinical hypothyroidism (n=17) shows 17 fully tested (65.4%), 4 partially tested (15.4%), and 5 not tested (19.2%) (Supplementary Figure S1).

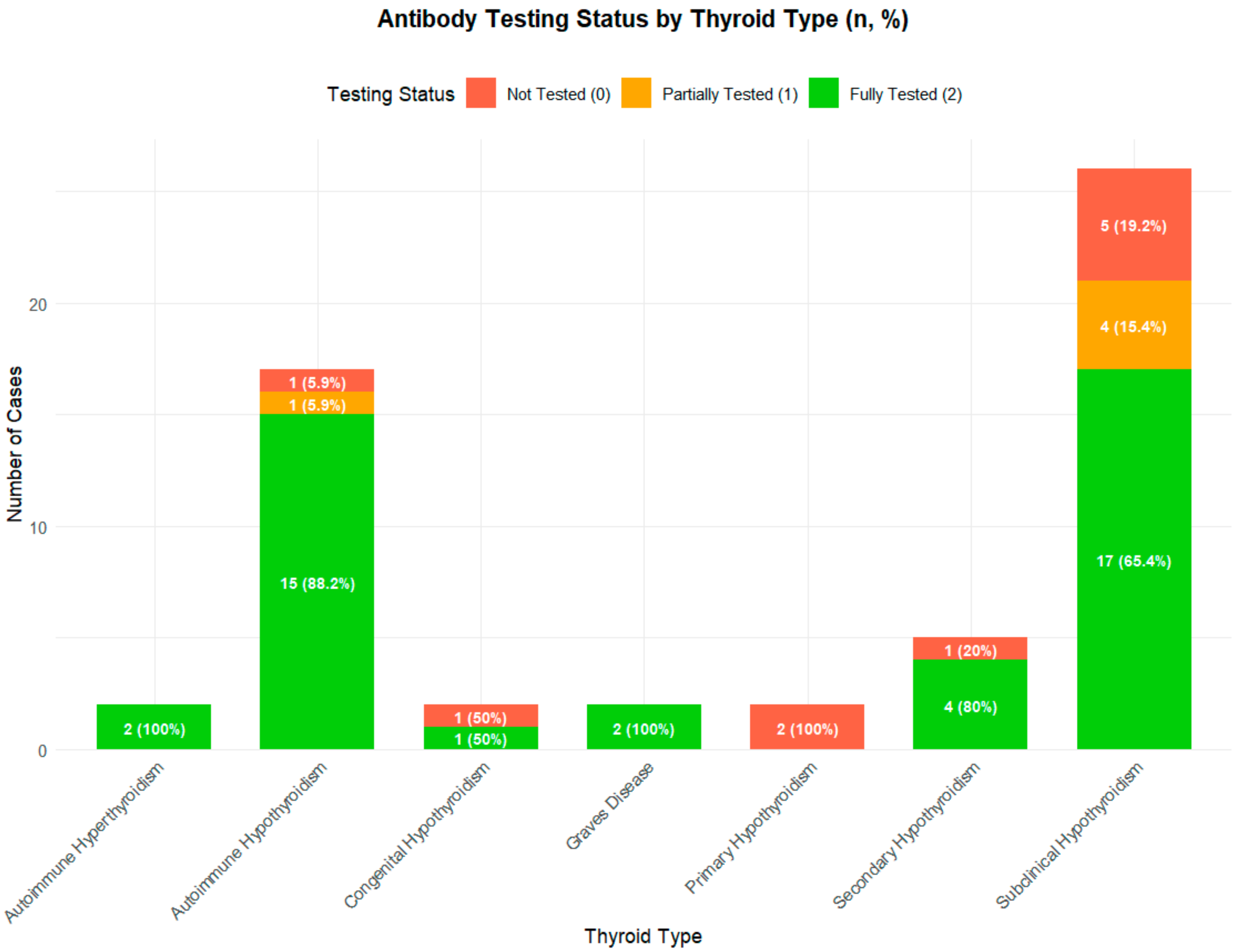

Supplementary Figure S1: Number and percentage of patients with thyroid disease tested (partially or fully) or no tested for Anti-TPO and Anti-TG.

Anti-TG antibody testing patterns revealed expected findings: 100% positivity in autoimmune hyperthyroidism and Graves' Disease, 70.6% positivity in autoimmune hypothyroidism, and 23.1% positivity in subclinical hypothyroidism. Testing was notably incomplete in several groups: 34.6% of subclinical hypothyroidism cases and 50% of congenital hypothyroidism cases were not tested for Anti-TG antibodies (Supplementary Table S3).

Supplementary Table S3: Anti-Thyroglobulin (Anti-TG) Antibody Status by Thyroid Dysfunction Type.

**Thyroid Dysfunction Type**	Anti-TG Positive n (%)	Anti-TG Negative n (%)	Not Tested n (%)
Autoimmune hyperthyroidism (n=2)	2 (100.0%)	0 (0.0%)	0 (0.0%)
Graves' disease (n=2)	2 (100.0%)	0 (0.0%)	0 (0.0%)
Autoimmune hypothyroidism (n=17)	12 (70.6%)	3 (17.6%)	2 (11.8%)
Subclinical hypothyroidism (n=26)	6 (23.1%)	11 (42.3%)	9 (34.6%)
Congenital hypothyroidism (n=2)	0 (0.0%)	1 (50.0%)	1 (50.0%)
Primary hypothyroidism (n=2)	0 (0.0%)	0 (0.0%)	2 (100.0%)
Secondary hypothyroidism (n=5)	0 (0.0%)	4 (80.0%)	1 (20.0%)

TPO antibody testing was 100% positive in autoimmune hyperthyroidism, 94.1% in autoimmune hypothyroidism, and 65.4% in subclinical hypothyroidism. The substantial TPO positivity in subclinical hypothyroidism (65.4%) supports the autoimmune nature of many cases classified in this category (Supplementary Table S4).

Supplementary Table S4: Anti-Thyroid Peroxidase (TPO) Antibody Positivity by Thyroid Dysfunction Type in Children with Down Syndrome.

Thyroid Dysfunction Type	TPO Positive n (%)	TPO Negative n (%)	Not Tested n (%)
Autoimmune Hyperthyroidism (n=2)	2 (100.0%)	0 (0.0%)	0 (0.0%)
Autoimmune Hypothyroidism (n=17)	16 (94.1%)	0 (0.0%)	1 (5.9%)

Subclinical Hypothyroidism (n=26)	17 (65.4%)	4 (15.4%)	5 (19.2%)
Congenital Hypothyroidism(n=2)	0 (0.0%)	1 (50.0%)	1 (50.0%)
Graves Disease(n=2)	0 (0.0%)	2 (100.0%)	0 (0.0%)
Primary Hypothyroidism (n=2)	0 (0.0%)	0 (0.0%)	2 (100.0%)
Secondary Hypothyroidism (n=5)	0 (0.0%)	4 (80.0%)	1 (20.0%)

Among tested patients, any antibody positivity (Anti-TPO, Anti-TG) was 100% and 94.1% of autoimmune hyperthyroidism and hypothyroidism, respectively. In subclinical hypothyroidism, positivity ranged from 50 to 100% depending on DS karyotype. Notably, among the 23 trisomy 21 patients with subclinical hypothyroidism, 65.2% had any antibody positive, and 17.4% had both antibodies positive (Supplementary Table S5).

Supplementary Table S5: Thyroid Autoantibody Positivity by Thyroid Dysfunction Type and Down Syndrome Karyotype.

Thyroid Dysfunction Type	Down Syndrome Type	n	Any Antibody Positive n (%)	Both Antibodies Positive n (%)	TPO Positive n (%)	TG Positive n (%)
Autoimmune Hyperthyroidism	Trisomy 21	2	2 (100.0%)	2 (100.0%)	2 (100.0%)	2 (100.0%)
Autoimmune Hypothyroidism	Trisomy 21	17	16 (94.1%)	12 (70.6%)	16 (94.1%)	12 (70.6%)
Subclinical Hypothyroidism	Trisomy 21	23	15 (65.2%)	4 (17.4%)	15 (65.2%)	4 (17.4%)
Subclinical Hypothyroidism	Translocation	1	1 (100.0%)	1 (100.0%)	1 (100.0%)	1 (100.0%)
Subclinical Hypothyroidism	Mosaic	2	1 (50.0%)	1 (50.0%)	1 (50.0%)	1 (50.0%)
Graves Disease	Trisomy 21	2	2 (100.0%)	0 (0.0%)	0 (0.0%)	2 (100.0%)

Congenital Hypothyroidism	Translocation	2	0 (0.0%)	0 (0.0%)	0 (0.0%)	0 (0.0%)
Primary Hypothyroidism (n=2)	Trisomy 21	2	0 (0.0%)	0 (0.0%)	0 (0.0%)	0 (0.0%)
Secondary Hypothyroidism	Trisomy 21	5	0 (0.0%)	0 (0.0%)	0 (0.0%)	0 (0.0%)

Among subclinical hypothyroidism, 6 cases were not tested, 3 cases were partially tested while among autoimmune hypothyroidism one case was not tested and another case was not fully tested.

Within the thyroid dysfunction cohort, Trisomy 21 patients showed the highest rates of antibody positivity: 64.7% TPO positive and 39.2% TG positive. Translocation and mosaic patients showed lower positivity rates, but sample sizes were small (n=3 and n=2, respectively). Testing completeness varied: 15.7% of trisomy 21 patients, 33.3% of translocation patients, and 50.0% of mosaic patients were not tested for TPO antibodies (Supplementary Table S6).

Supplementary Table S6 : Thyroid Autoantibody Status by Down Syndrome Karyotype.

Down Syndrome Type	n	TPO Positive n (%)	TPO Negative n (%)	TPO Not Tested n (%)	TG Positive n (%)	TG Negative n (%)	TG Not Tested n (%)
Mosaic (n=2)	2	1 (50.0%)	0 (0.0%)	1 (50.0%)	1 (50.0%)	0 (0.0%)	1 (50.0%)
Translocation (n=3)	3	1 (33.3%)	1 (33.3%)	1 (33.3%)	1 (33.3%)	1 (33.3%)	1 (33.3%)
Trisomy 21 (n=51)	51	33 (64.7%)	10 (19.6%)	8 (15.7%)	20 (39.2%)	18 (35.3%)	13 (25.5%)

Among the 41 patients on levothyroxine, dosing was adequate in only 26.8% overall. The problem was most severe in secondary hypothyroidism (100% inadequate, n=5) and substantial in both autoimmune hypothyroidism (70.6% inadequate) and subclinical hypothyroidism (75.0% inadequate). Mean daily doses ranged from 20.0 mcg for subclinical hypothyroidism to 150.0 mcg for congenital hypothyroidism, providing context for treatment intensity across disorders (Supplementary Table S7).

Supplementary Table S7: Treatment Adequacy by Thyroid Dysfunction Type.

Thyroid Disorder Type	Total N	On Therapy, n	Mean Dose, mcg/day	Dosing Adequate, n (%)	Doing Inadequate, n (%)
Autoimmune Hypothyroidism	17	17	53.7	5 (29.4)	12 (70.6)
Subclinical Hypothyroidism	26	16	20.0	4 (25.0)	12 (75.0)
Secondary Hypothyroidism	5	5	62.5	0 (0.0)	5 (100.0)
Congenital Hypothyroidism	2	2	150.0	1 (50.0)	1 (50.0)
Primary Hypothyroidism (n=2)	2	1	125.0	1 (100.0)	0 (0.0)

Predictors of thyroid disease among patients with DS. Family History of Thyroid Disease: This factor demonstrates the strongest link, with an adjusted odds ratio (aOR) of 4.57 and a 95% confidence interval (CI) ranging from 1.89 to 11.6. Total Symptoms: With an aOR of 1.92 (95% CI: 1.22 to 3.25). This means that with each additional symptom, the likelihood of having thyroid dysfunction rises by 92% (Supplementary Figure S2).

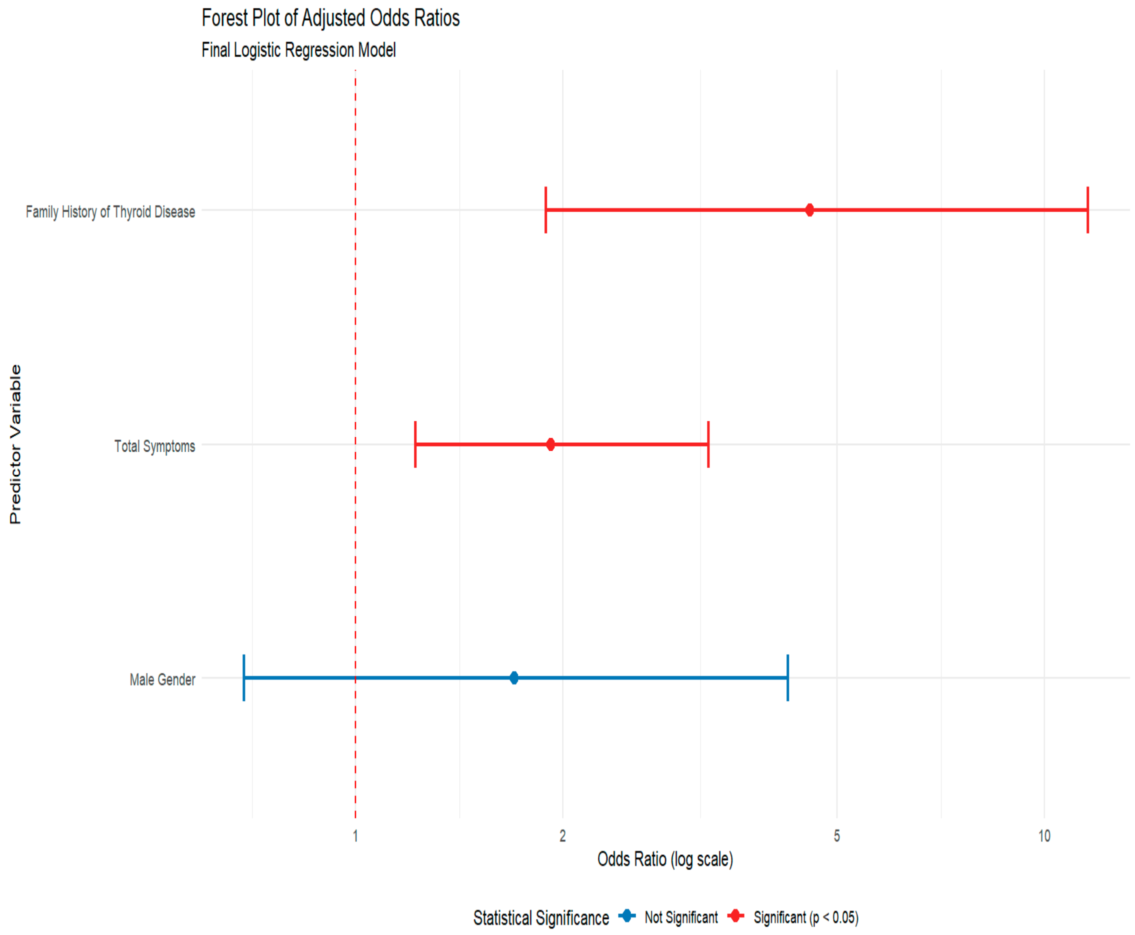

Supplementary Figure S2: Forest plot of the predictors of having thyroid dysfunction among patients with DS.
